# Mechanical Tension Drives Elongational Growth of the Embryonic Gut

**DOI:** 10.1038/s41598-018-24368-1

**Published:** 2018-04-16

**Authors:** Nicolas R. Chevalier, Tinke-Marie de Witte, Annemiek J. M. Cornelissen, Sylvie Dufour, Véronique Proux-Gillardeaux, Atef Asnacios

**Affiliations:** 1 Laboratoire Matière et Systèmes Complexes, Université Paris Diderot/CNRS UMR 7057, Sorbonne Paris Cité, 10 rue Alice Domon et Léonie Duquet, 75013 Paris, France; 2 INSERM, U955, Equipe 06, 94000 Créteil, France; 3 Université Paris Est, Faculté de médecine, 94000 Créteil, France; 4 Institut Jacques Monod, UMR 7592, CNRS & Univ. Paris Diderot, 15 Rue Hélène Brion, 75013 Paris, France

## Abstract

During embryonic development, most organs are in a state of mechanical compression because they grow in a confined and limited amount of space within the embryo’s body; the early gut is an exception because it physiologically herniates out of the coelom. We demonstrate here that physiological hernia is caused by a tensile force transmitted by the vitelline duct on the early gut loop at its attachment point at the umbilicus. We quantify this tensile force and show that applying tension for 48 h induces stress-dependent elongational growth of the embryonic gut in culture, with an average 90% length increase (max: 200%), 65% volume increase (max: 160%), 50% dry mass increase (max: 100%), and 165% cell number increase (max: 300%); this mechanical cue is required for organ growth as guts not subject to tension do not grow. We demonstrate that growth results from increased cell proliferation when tension is applied. These results outline the essential role played by mechanical forces in shaping and driving the proliferation of embryonic organs.

## Introduction

The intestine is the body’s longest organ with a length of 6 m on average in the human adult^1^, and the most elongated, as its diameter is only ~2.5 cm. This very high aspect ratio results from strong, anisotropic growth during the embryonic, fetal and neonatal period. In recent years, mechanical forces have been found to have a profound influence on the development of several organs including the lung^2^, heart^3^, kidney^4^, joint^5^; recent studies have shown that mechanical buckling drives fetal gut looping^6^ and epithelial villus formation^7,8^. We question here the influence of mechanical forces on embryonic gut overall growth and shape.

## The Embryonic Gut is Physiologically under Mechanical Tension

**Figure 1:**
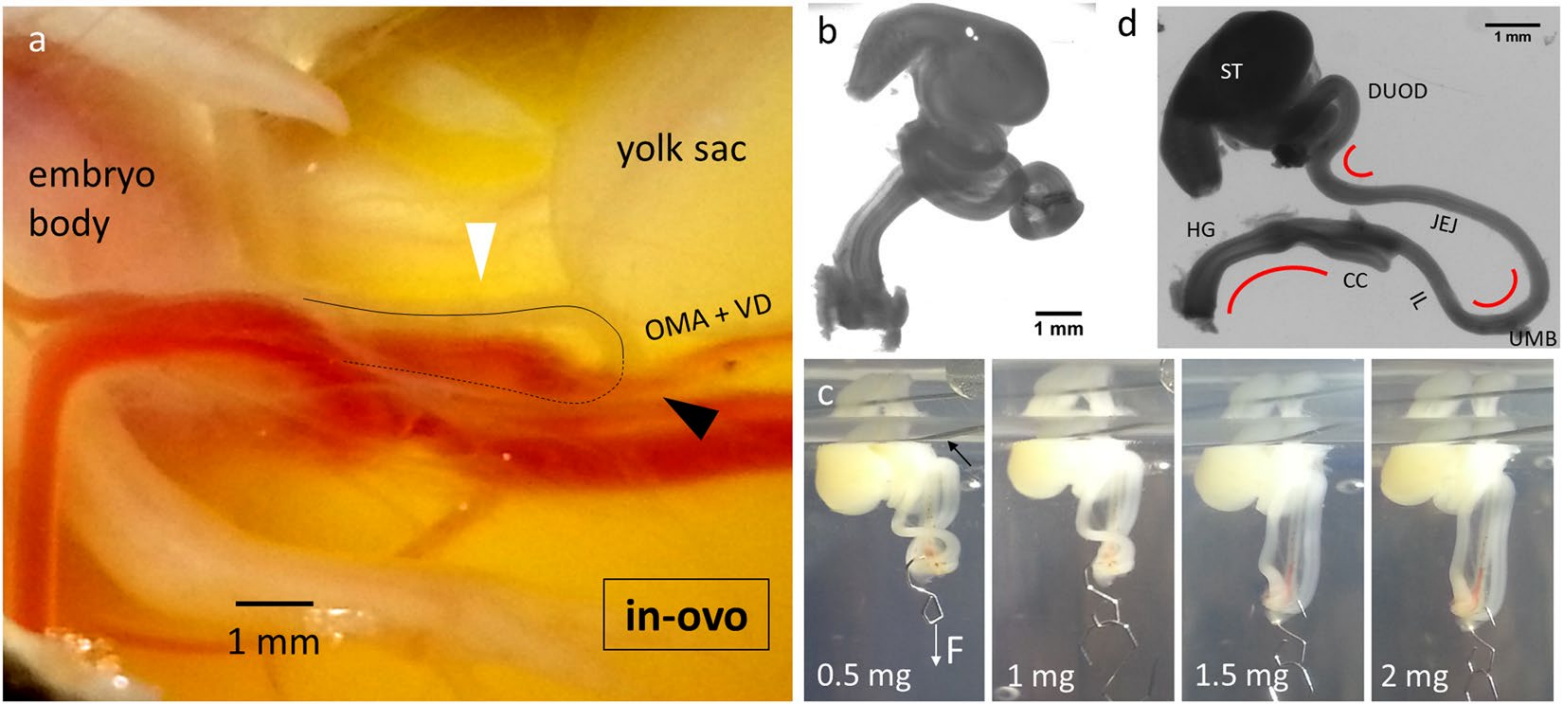
The embryonic gut loop is in a state of longitudinal mechanical tension. (**a**) Top-view of E9 chicken embryo *in-ovo*, lying on its side. Black line: midline of the gut (white arrowhead: jejunum). The gut herniates out of the body, through the umbilical cord. Black arrowhead: attachment at the umbilicus of the omphalomesenteric artery (OMA) and vitelline duct (VD), which extend to the right into the yolk sac. (**b**) The gut (E8) spontaneously coils up when tensile forces are released after dissection. (**c**) Measurement of the force required to uncoil the gut. The black arrow points at the upper pin that holds the stomach and hindgut. (**d**) After removal of the mesentery, three permanent bends (red curved lines) are visible at the level of the hindgut, umbilicus and jejunum-duodenum junction. ST: stomach, DUOD: duodenum, JEJ: jejunum, UMB: umbilicus, IL: ileum, CC: caecal appendix, HG: hindgut.

**Figure 2:**
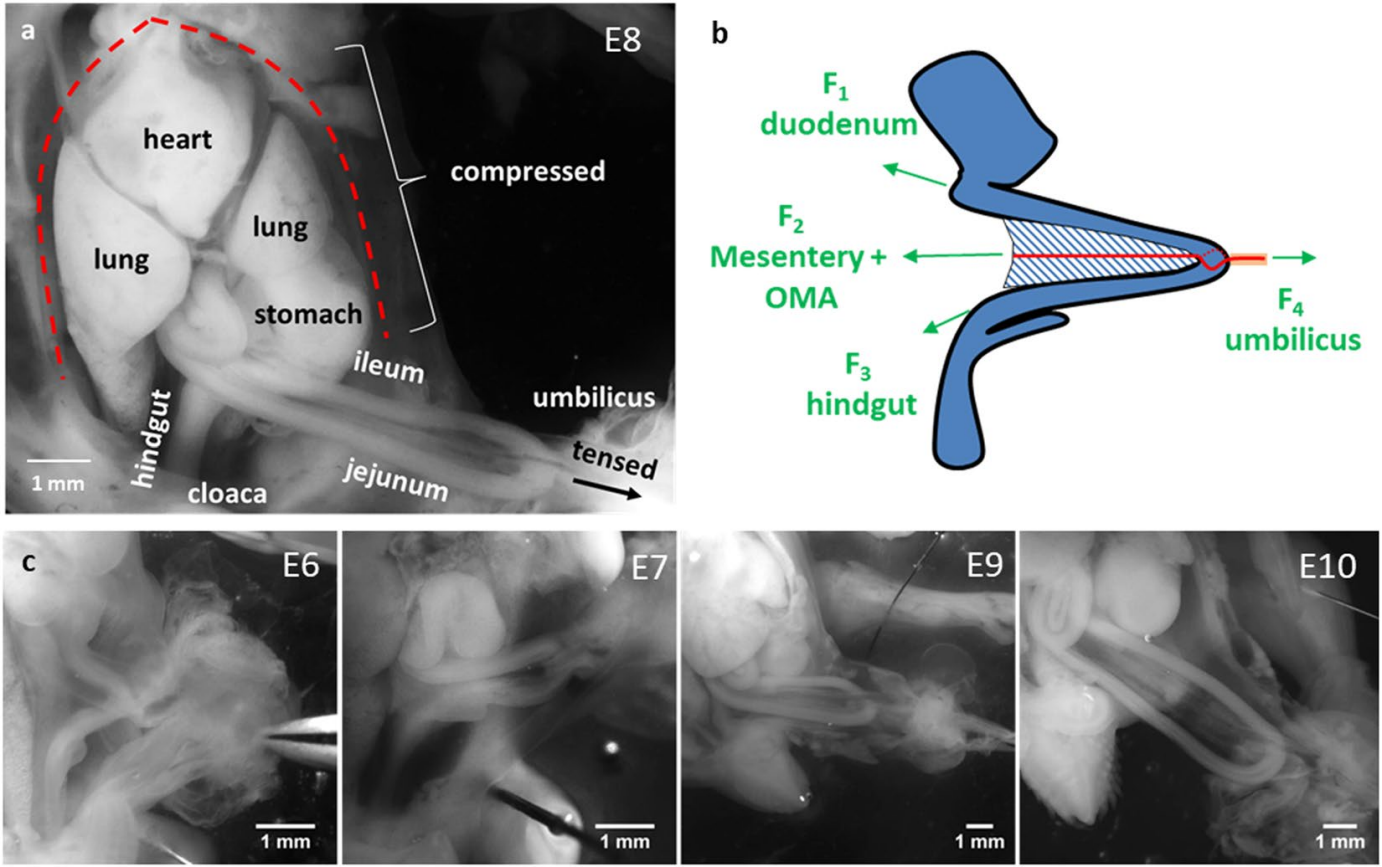
Mechanical configuration of embryonic organs. (**a**) E8 chick embryo after removing the ventral skin of the body. The heart, lungs, stomach and liver grow compressed one against another and within the limited space of the body cavity (red dashed line). The gut is singular as it protrudes out through the umbilical cord and can expand freely outside of the embryo’s body. (**b**) Free-body diagram of the embryonic gut, midgut rotation was not represented for visual clarity. Green: tensile forces F_1_-F_4_, dashed region: mesentery, red: omphalomesenteric artery, beige: vitelline duct. (**c**) Geometry of the gut at E6, E7, E9, E10. An opening was performed on the left side of the ventral skin to reveal internal organs. In this figure the gut was pulled slightly to the right with tweezers via the vitelline duct to show it in a close to physiological configuration (Fig. 1a).

Figure 1a shows a photograph of an E9 chicken embryo *in-ovo*, lying on its side. The gut loop (black line and white arrowhead in Fig. 1a) forms a relatively straight “U” that protrudes through the umbilical cord out of the embryo’s body, a situation referred to as “physiological umbilical hernia”^9,10^. Gut hernia has been attributed to excessive growth of the liver^9^ or of the gut itself, causing it to bulge out into the coelom. The rostral (stomach) and caudal (hindgut) ends of the gut are attached by conjunctive tissue to the inner dorsal wall of the body cavity. A third attachment point is provided at the level of the umbilicus (the turn of the “U”) by the vitelline duct and accompanying omphalomesenteric artery (OMA). The vitelline duct connects the midgut with the yolk sac; the connection is secured by the omphalomesenteric artery which forms a ring around the gut (Fig. 1a black arrowhead). When we cut the connection of the gut loop to the vitelline duct and omphalomesenteric artery at the level of the umbilicus, the gut progressively (within ~10 min) coiled up (Fig. 1b), forming loops that were not present *in-ovo* at this stage (Fig. 1a). The formation of these 3D loops proceeds by buckling^6^. It shows that tension was present in the gut and mesentery, and was released when the attachments (vitelline duct + omphalomesenteric artery) to the yolk sac were cut. We measured how much tension is exerted by fixing the stomach and hindgut of isolated (coiled up) E7.5-E8 embryonic guts to a pin, and by incrementally attaching small 0.5 mg weights at the level of the umbilicus as shown in Fig. 1c. Measurements were performed in PBS at room temperature. The morphology of the gut was evaluated at least 1 h after the weight was fastened to the umbilicus, so it reached a stationary state. We found that guts remained coiled at 0 mg (no weight, n = 3) and 0.5 mg (n = 3). At 1 mg, one sample uncoiled, whereas 4 samples remained coiled. The necessary mass to uncoil the remaining 4 samples was 1.5–2 mg. An overall estimate is therefore 1–2 mg, which after correcting for buoyancy corresponds to a minimal tensile force applied on the early gut loop of 9–17 μN (Explanatory Note S1). This stress is distributed on the two midgut branches (jejunum and ileum), the mesentery, and the OMA. The stress acting on the mesentery membrane located between the two gut branches and the OMA is transmitted directly to the dorsal wall; it does not contribute to stretching of the gut. To isolate this contribution we measured the resistance to stretch of the isolated mesentery + OMA and compared it to that of the two midgut branches (Fig. S2). We find that the mesentery + OMA absorbs ~25% of the total force transmitted via the vitelline duct; each gut branch is therefore subject to a minimal tension of 3.4–6.6 μN. We additionally observe that the embryonic gut presents three physiologic permanent bends at the level of the hindgut, umbilicus and duodenum (Fig. 1d, red curves) which are characteristic of an organ which has been irreversibly bent because of chronic stretch by the forces depicted in Fig. 2b. From stages E6 through E10, the two arms of the “U” (the jejunum and the ileum) grow to about the same length (Fig. 2a,c): the geometry of the gut loop is therefore consistent with the free-body diagram depicted in Fig. 2b. All these observations point to the fact that the early gut (E6-E10) is in a state of mechanical tension and can freely grow outside of the embryo’s body. In contrast, other organs (heart, lungs, stomach) grow compressed one against the other inside the body cavity of the embryo (Fig. 2a) resulting in a 3D “jigsaw puzzle” in which every organ’s external surface smoothly fits the surface of its neighboring organ. As from E11-E12, additional gut loops start forming *in-ovo*^10^ and the simple hairpin geometry depicted in Fig. 2b is no longer valid. In humans, the hairpin configuration is an accurate description from CS14 to CS18^11–13^.

## Mechanical Tension Induces Volumetric Growth and Elongation of the Gut in Culture

**Figure 3:**
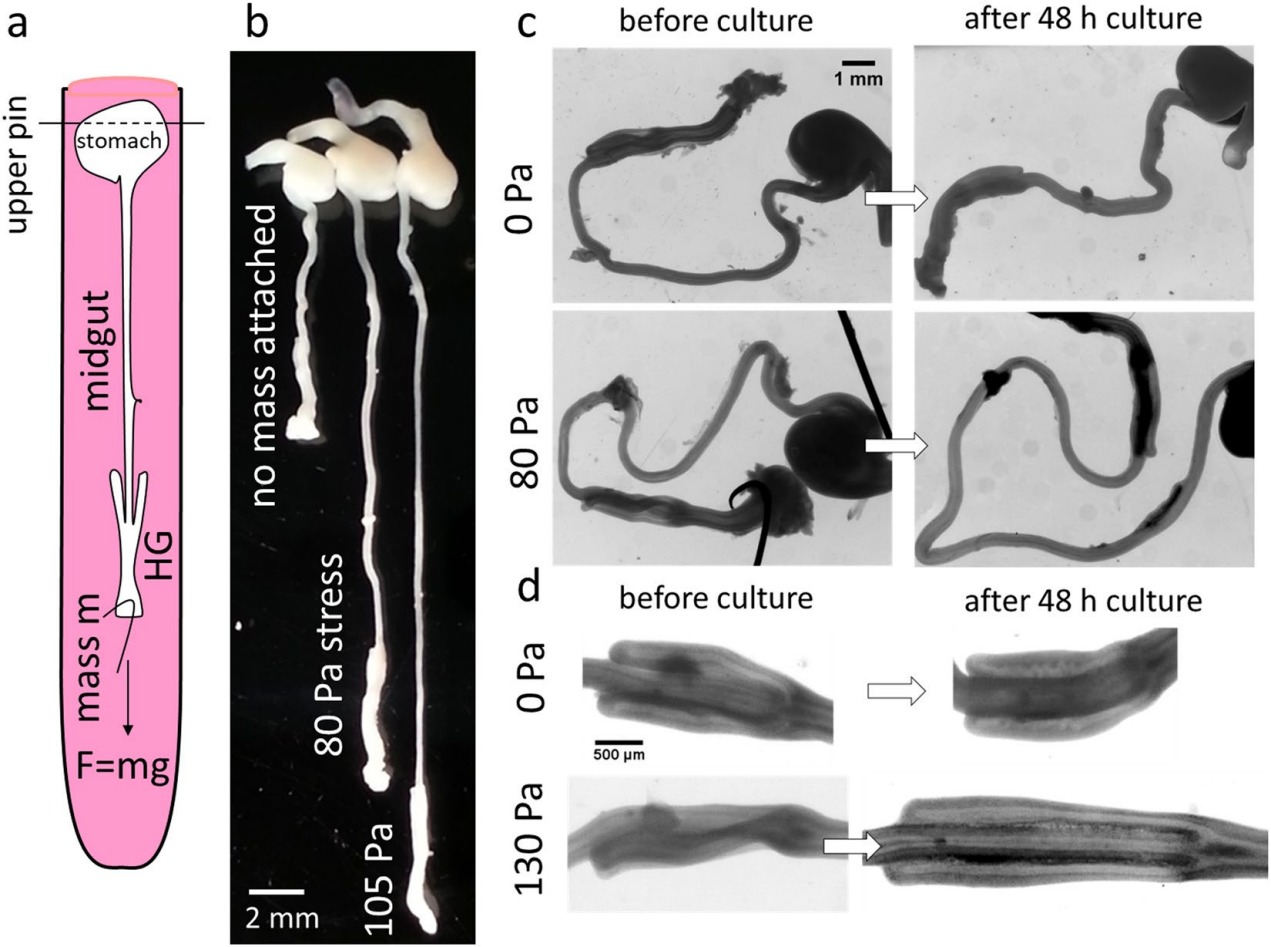
Static mechanical tension induces growth of cultured guts. (**a**) Scheme of culture method to apply static longitudinal mechanical stress (see Materials & Methods). (**b**) Representative outcome of an experiment with 3 E8 guts cultured respectively without stress and with 80 Pa and 105 Pa applied stress, after 48 h culture. (**c**,**d**) Before/after 48 h culture comparison of whole gut (**c**) and caeca (**d**) morphological changes without (top) and with (bottom) tension. The scale of the whole guts (**c**) or caeca (**d**) is that shown in the top left quadrant.

**Figure 4:**
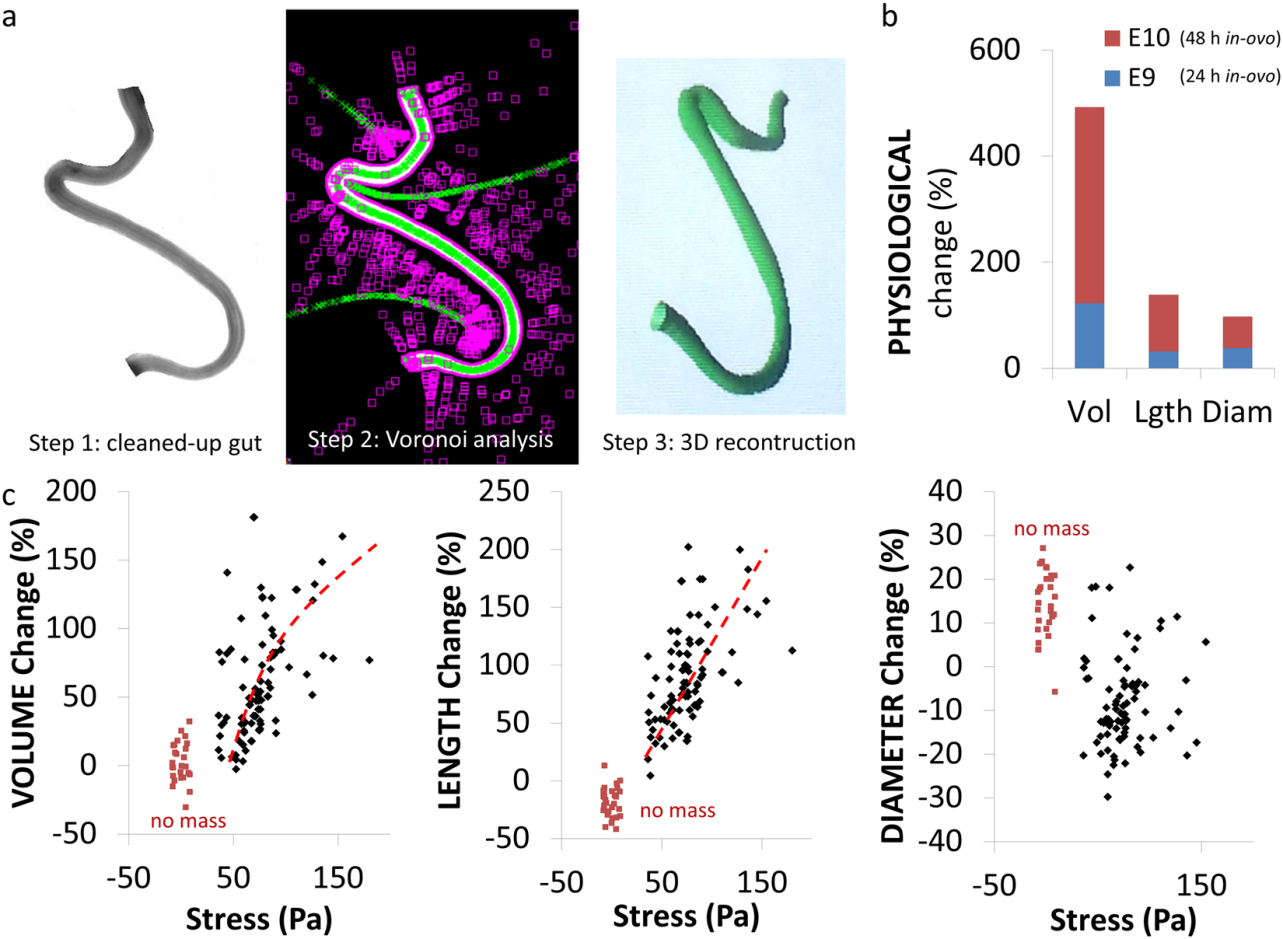
Quantitative morphometric changes *in-ovo* and in culture. (**a**) From a 2D photograph of the demesenterized midgut (step 1), a Voronoi tessellation algorithm extracts gut axis and contour (step 2), allowing to retrieve gut length, average diameter and volume (step 3: 3D reconstruction using the Olaf ImageJ plugin)^43^. (**b**) Physiological *in-ovo* volume, length and diameter changes at E9 and E10 relative to the state at E8 (*n* = 3 for each stage). (**c**) Volume, length and diameter changes induced by 48 h culture without tension (red squares, *n* = 27) or with tension (black diamonds, *n* = 95) as a function of the stress applied by the hanging mass (see Explanatory Note S3). Red dashed lines are trend guides. Each data point corresponds to a different gut. The data are the result of 21 independent experiments.

We next questioned whether this tensile force played a role in the development and growth of the embryonic gut. Hearn *et al*.^14^ recognized the importance of the physical state of the gut in *ex-vivo* culture: they developed a setup (catenary culture) that reproduces the “free-floating” configuration of the gut *in-vivo*. This setup does not however apply tension continuously. It allows for tubular morphogenesis of the organ in culture, but growth was slight and limited to the caecal region. Here, we developed a new *ex-vivo* culture method to apply continuous mechanical tension by gravity (Fig. 3a, Materials & Methods). After 48 h culture, control guts cultured without tension appeared slightly shorter and thicker than they initially were (Fig. 3b,c); guts cultured with a 1 mg mass (~70–100 Pa applied stress, corresponding to ~7–10% instantaneous elastic strain, Explanatory Note S3) had grown to a considerable length (Fig. 3b,c); 1.5 mg tension (100–140 Pa, ~10–14% instantaneous elastic strain) led to strong elongation but also to thinning (Fig. 3b). Applying tension also induced significant caecal appendix growth (Fig. 3d). To quantify midgut morphological (volume, length, diameter) changes, we developed software relying on Voronoi tessellation and 3D reconstruction (Fig. 4a, Materials & Methods). The initial diameter of the dissected guts varied from sample-to-sample. We therefore present the morphological changes in Fig. 4b,c as a function of the applied initial mechanical stress *σ* on each gut $\sigma =F/S$, where *F* is the force applied by the hanging mass (Explanatory Note S1), and $S=\pi {d}^{2}/4$ is the initial cross section of each individual gut, with *d* the initial diameter (before culture) of each individual gut.

Control guts decreased in length on average by 18 ± 6% (*n* = 27); this decrease in length was accompanied by an increase in diameter of 14 ± 7%; overall the volume of the control guts did not change (+2 ± 7%). Applying tension led to an increase in length of the gut that was proportional to the applied stress (between +50 and +200% in the 50–150 Pa stress range). The volume of the tensed guts also increased with applied stress (between 0 and +180% in the 50–150 Pa applied stress range). A volume change indicates an active biological response of the gut to the application of mechanical stress; passive viscoelastic deformation of the gut alone would not have resulted in any net volume change. The diameter of the tensed guts was on average smaller after culture (−7% ± 6%, *n* = 95). Although diameter tended to decrease at higher stress values (Fig. 3b), diameter change did not exhibit a clear-cut correlation with applied stress (Fig. 4c). This is due to the fact that high stresses induced both viscoelastic lengthening and thinning of the gut, but that they also triggered an active biological volume increase that partially counterbalanced viscoelastic thinning. Comparing the physiological morphological changes *in-ovo* (Fig. 4b) over a 48 h period to those induced by culture under tension (Fig. 4c), we see that the length changes in culture (50–200%) and *in-ovo* (~110%) are in the same range, but that the volume increase is much smaller in culture (0–180%) than *in-ovo*^15–17^ (~500%). This is not surprising as the cultured guts lack many components of physiological growth such as hormones and blood supply. These are clearly important factors: guts grown on the chorio-allantoic membrane, where vascularization is re-established after the organ has been dissected out, have important growth rates^15,18,19^. Whereas the diameter of the gut increases *in-ovo* (~60%) we found that it tended to decrease in our culture experiment (−7%). From the irreversible elongation of the midgut ${\rm{\Delta }}l/l$ over the culture time ${\rm{\Delta }}T=48\,{\rm{h}}$ and the longitudinal stress σ we can deduce an effective tissue viscosity $\eta =\frac{\sigma {\rm{\Delta }}T}{{\rm{\Delta }}l/l}\sim {10}^{9}$ Pa.s. This value is 10^4^ times higher than the short-time (~1 h) passive mechanical viscosity of cell aggregates^20^; it is of the same order of magnitude as the effective viscosity due to cell proliferation over a 24 h period in aggregates^21,22^. Volumetric growth of the ileum was comparable to that of the jejunum (Fig. S4); caecal appendix growth (Fig. S5) presented similar characteristics to that of the midgut.

## Increased Cell Proliferation Underlies the Volume and Mass Increase of Tensed Guts

**Figure 5:**
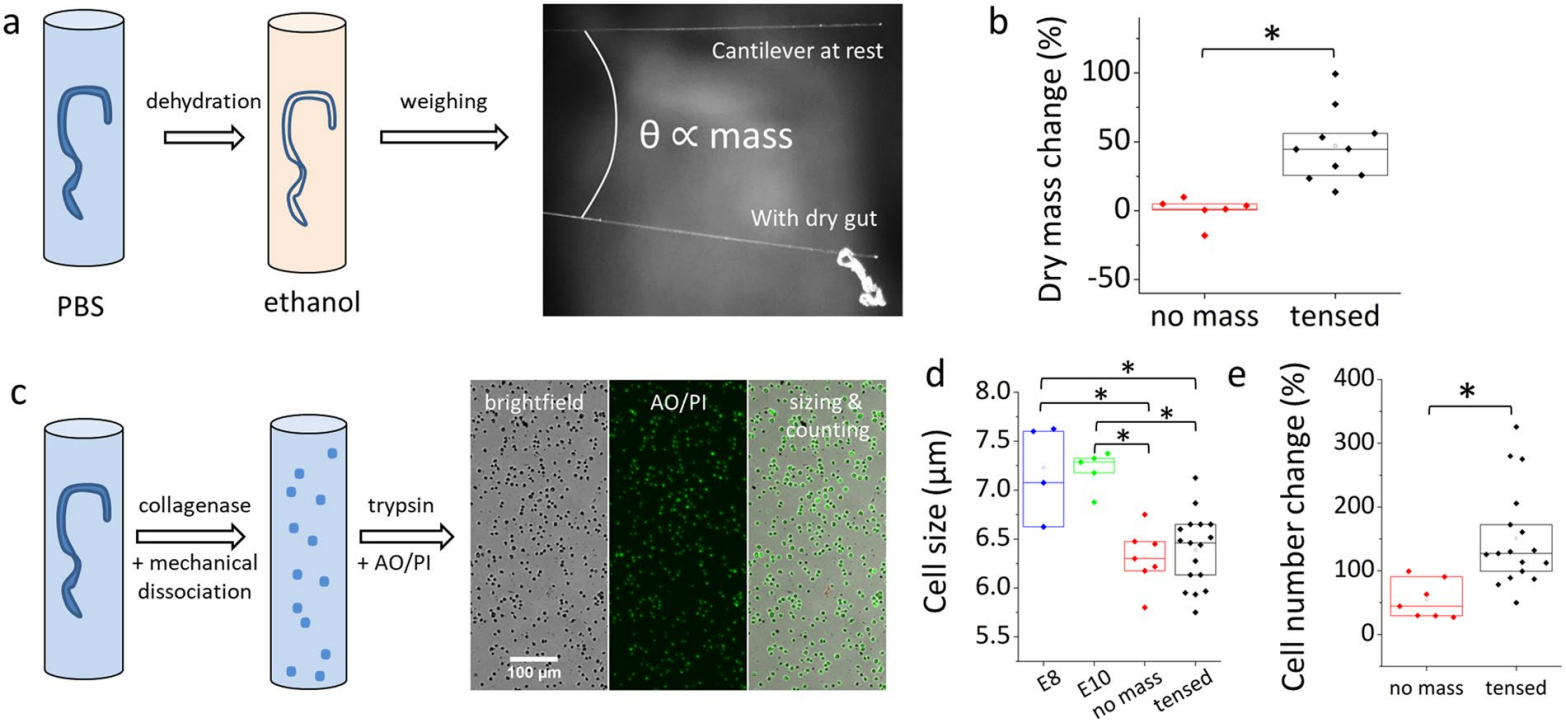
Dry mass, cell size and cell number changes of cultured guts. Data of each figure is the result of at least 3 independent experiments, each point corresponds to a separate sample (gut). (**a**) Scheme of the method to measure dry mass (see Materials & Methods, Video S6, Figs S7, 8). (**b**) Dry mass variation of E8 guts cultured without (red squares, *n* = 6) or with (black squares, *n* = 10) applied tension. The stress applied was in the range 50–150 Pa. (**c**) Scheme of gut dissociation protocol and resulting brightfield, acridine orange/propidium iodide (AO/PI) and analyzed image of cells in counting chamber (see Materials & Methods). (**d**) Average measured cell size in E8 guts cultured without (*n* = 7) or with (*n* = 17) applied tension, and for native E8 (blue squares, n = 4) and E10 guts (green dots, n = 5). The stress applied was in the range 50–150 Pa. (**e**) Cell number change in E8 guts cultured without (*n* = 7) or with (*n* = 17) applied tension. *p < 0.05, Mann Whitney two-tailed test.

We next sought to determine whether the volume increase of the guts cultured with tension led to an increase of the organ’s dry mass. Cultured guts were dehydrated and weighed with a fiber cantilever (Fig. 5a, Video S6, Figs S7, 8, Materials & Methods); the dry mass before culture was deduced from its volume (see Materials & Methods). We found that the dry mass (Fig. 5b) of the guts cultured without tension did not change (0.3 ± 9.6%, *n* = 6). Applying tension (stress in the range 50–150 Pa) led to a significant dry mass increase (47 ± 26%, *n* = 10, Fig. 5b).

An increase in dry mass and volume could either be due to cell proliferation or to an increase of the volume of each cell within the organ (and of their protein and lipid content). To discriminate between these two possibilities, we counted the total number of cells in the organ after dissociation (see Figs 5c, S8b, Materials & Methods). Average cell size of cultured guts was smaller than for E8 or E10 guts (Fig. 5d). The reduction in cell size was similar for guts cultured with or without tension (Fig. 5d). This indicates that the volume and dry mass increase induced by tension is not due to single cell size increase. The total cell number change of guts cultured with tension was significantly higher (124 ± 96%, *n* = 17, Fig. 5e) than for control guts (55 ± 30%, *n* = 7). These results unambiguously demonstrate that the increase in gut volume and dry mass observed when the guts are subject to mechanical tension for 48 h is the result of a net increase in cell number. This increase in cell number with applied tension is ~4 times lower than the physiological cell number change over a 48 h period (~500% based on the volume data of Fig. 4b and on the fact that cell size is constant between E8 and E10, Fig. 5d).

**Figure 6:**
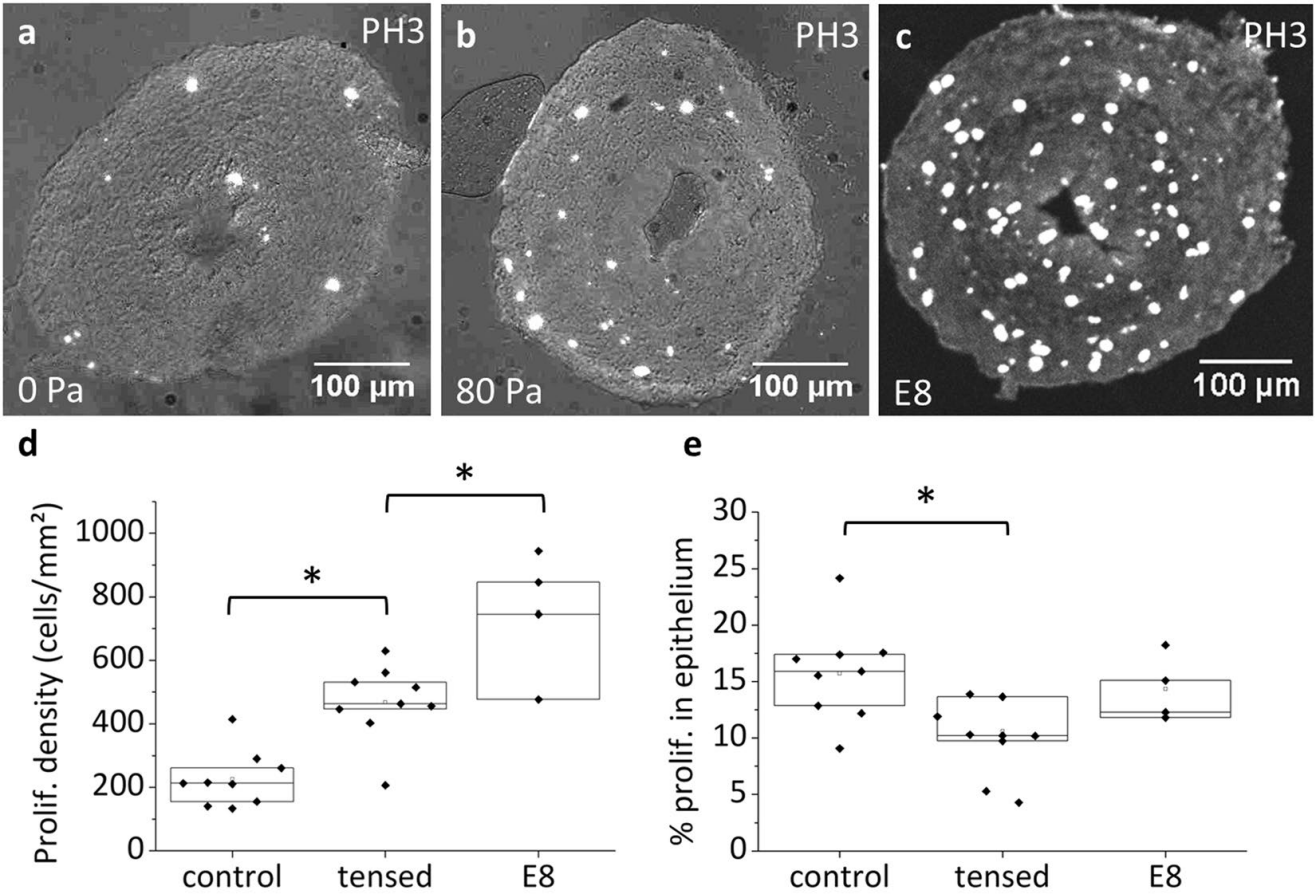
Proliferation in cultured and native E8 guts. Phospho-histone S10 immunohistochemical staining of midgut section of E8 gut cultured for 48 H without tension (**a**), with tension (**b**) and for a native, uncultured E8 gut (**c**). Proliferating cells appear as GFP bright spots, overlaid over the brightfield image (gray). (**d**) Proliferation density (number of PH3 positive cells on a section divided by the section surface area) for *n* = 9 unweighed, *n* = 9 weighed (1 mg, stress in the range 70–140 Pa) and *n* = 4 uncultured E8 guts. The average proliferation density for each sample is obtained from *n* = 6–18 different slices. (**e**) Percentage of proliferating cells found in the epithelium. *p < 0.05, Mann Whitney two-tailed test.

Figure 6 shows the proliferation rate (anti-histone H3 phospho S10 antibody) of guts cultured without and with tension after 48 h culture, and of native, uncultured E8 guts. The average proliferation density (number of PH3 positive cells on a section divided by the section surface area) was 225 ± 88 cells/mm^2^ for unweighed guts (*n* = 9), 468 ± 119 cells/mm^2^ for weighed guts (70–140 Pa stress, *n* = 9) and 752 ± 201 cells/mm^2^ for native E8 guts. The presence of PH3 positive cells in unweighed guts (Fig. 6a,d) is consistent with the slight increase in cell number we measured (Fig. 5e). The significantly higher proliferation rates in tensed guts compared to controls is consistent with the idea that growth in the tensed guts results from an increased proliferation rate in response to mechanical stress. The proliferation rates of unweighed and weighed guts were both significantly lower than the proliferation rates of native E8 gut, in agreement with the fact that gut growth in culture is slower than physiological growth (Fig. 4b). We found proliferating cells scattered across all regions of the gut, endoderm (epithelium) and mesoderm (Fig. 6a–c); proliferation was not restricted to a particular gut layer. The percentage of proliferating cells in the epithelium of tensed guts (9.9 ± 3.3%) was lower than for controls (15.7 ± 4.2%) and native E8 guts (14.3 ± 3%).

## Cultured Guts are Motile and Display Normal Smooth Muscle and Enteric Neurons

**Figure 7:**
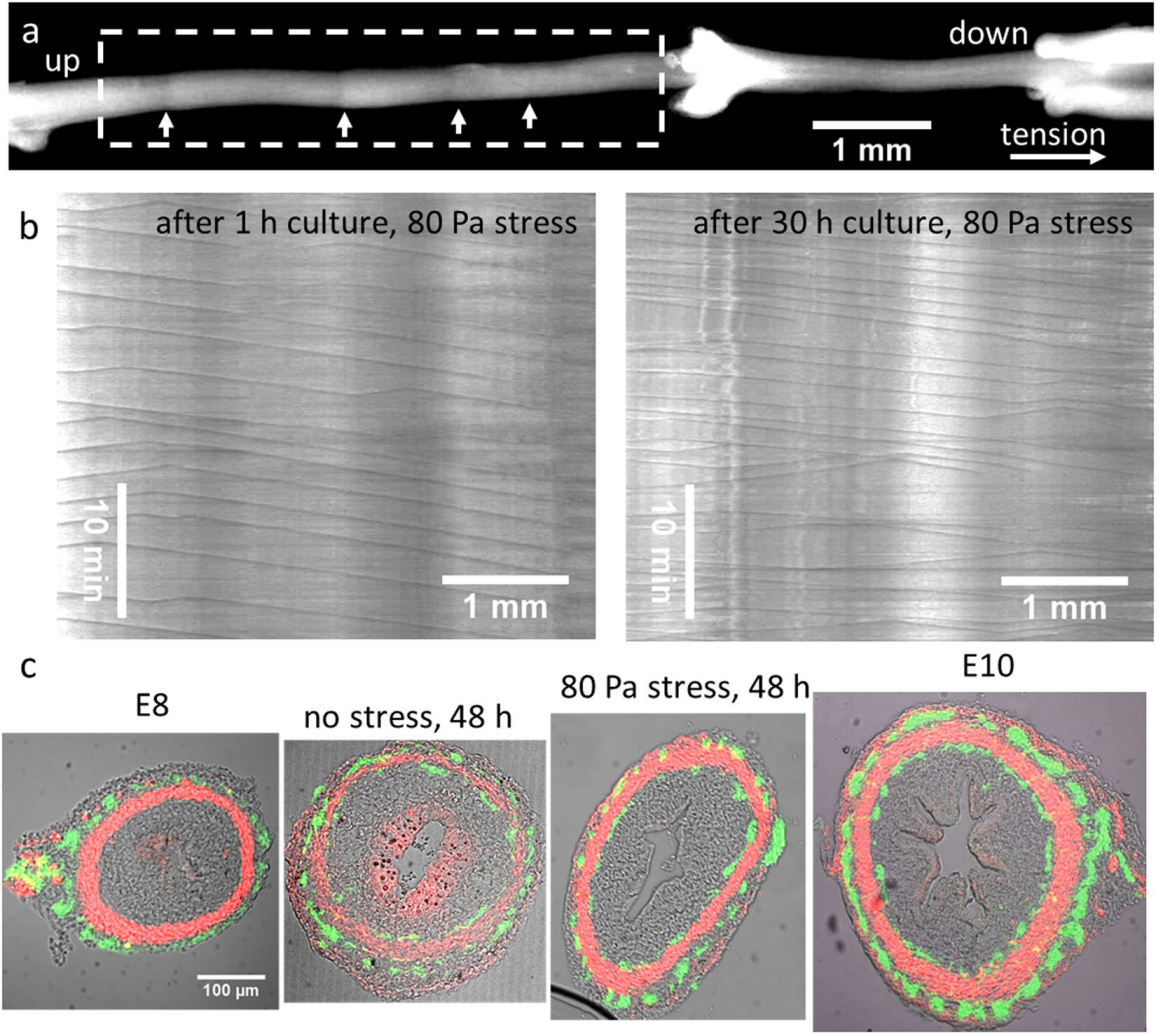
Cultured gut tissue histology and motility. (**a**) Frame from a time-lapse movie of motility during culture, applied stress: 80 Pa. The dark bands (white arrows) are left-to-right propagating constrictions of the smooth muscle (see Video S9). (**b**) Representative (*n* = 3) motiligrams (see Materials & Methods) derived from the region of interest indicated by a dashed rectangle in (**a**), 1 hour after the start of culture, and 30 hours later. The number of lines per unit time is the frequency of the contractile waves, the slope of each line yields their propagation speed^23^. (**c**) Midgut sections, green: βIII-tubulin (neurons), red: α-actin (smooth muscle), grey: brightfield, scale bar is the same for all four sections. Native E8 and E10 gut sections are also shown for comparison.

We found that the culture method we used resulted in active, living embryonic organs. Cells in the cultured guts were living as 99 ± 1% were green in response to AO/PI staining (Fig. 5c). During the whole culture period the guts exhibited spontaneous peristaltic activity (Fig. 7a,b)^23^. Peristaltic activity was present at the start of the experiment (Fig. 7b left and Video S9), after 30 h culture (Fig. 7b right), and after 48 h culture (Video S10). We further assessed histological features of the cultured organs (Fig. 7c). E8, E8+ 48 h culture (with or without tension) and E10 samples all exhibited a smooth muscle and a myenteric nerve layer; the submucosal plexus appeared distinctly on the E10 and the cultured gut sections. While the epithelium of the E10 gut had developed markedly compared to E8, forming distinct villi, it did not develop in our culture system. This is consistent with the fact that the percentage of proliferating cells found in the epithelium was lower in tensed guts than in native E8 guts (Fig. 6e). Proliferation of the epithelium in our culture system may be too slow compared to the longitudinal viscoelastic deformation & growth induced by the weights. It is likely that new epithelial cells are intercalated longitudinally, and therefore cannot give rise to the orthoradial buckling which is the first step of physiological villification in the chick gut^24^. We note that Walton *et al*. have recently suggested a different model for villification in the mouse based on a Turing reaction-diffusion model^25^.

## Discussion and Conclusion

Our results suggest that *in-ovo* mechanical tension transmitted by the vitelline duct and omphalomesenteric artery on the embryonic gut plays an important role in shaping this embryonic organ by driving proliferation, elongation (i.e., high aspect ratio cylindrical growth) and providing free space outside of the embryo body for the organ to grow unimpeded. We found that applying mechanical tension to the embryonic gut in culture is necessary to induce its growth; guts not subject to tension did not grow (no volume or dry mass increase, Figs 3b,c and 4c). The proliferative effect of stress on cell cultures has been reported^26–28^; we show here that this effect takes place at the scale of an embryonic organ, at similar values of applied elastic strain (5–15%, i.e., ~50–150 Pa stress). We moreover found that the growth rate of the organ increases with the applied elastic stress within the range 50–150 Pa (Fig. 4c). Such stress-dependent growth is well known for bone^29^ and plant cells^30^. These experimental data lend support to morphomechanical models and investigations relying on the assumption that the growth of soft animal tissues is stress-dependent^31,32^. They also show that embryonic development is flexible because it can dynamically respond to variations in mechanical loading. Reed *et al*.^33^ found that gut elongation in the zebrafish resulted from a longitudinal intercalation of radially migrating cells. Our results are consistent with this cell-scale view, as one expects longitudinal tensile forces acting on the gut tissue to precisely promote longitudinal intercalation of cells, thereby inducing elongation. It is noteworthy that applying mechanical tension to the neonate or adult gut has been shown to lead to irreversible lengthening and weight gain^34^, and is currently being studied to remedy Short Bowel Syndrome (SBS). The growth response to mechanical tension we investigated here may therefore apply not only to embryonic, but also to neonate and adult tissues.

We point at two limitations of our work. Modifying the tensile stress transmitted via the vitelline duct *in-ovo* would provide formal proof that tension-induced growth occurs during embryo development, and is not the result of a culture artefact. All our attempts at modifying stress in-ovo by mechanical manipulation have however failed because they led to hemorragias (the gut being located beneath vascularized membranes). We have shown that tension is physiologically transmitted to the gut, and believe it unlikely that mechano-biological proliferation effects observed in culture should be inhibited *in-ovo*. We also noticed that our culture method could not reproduce the physiological increase in diameter of the gut. Since a longitudinal stress induced longitudinal growth, it is reasonable to think that an orthoradial stress (caused for example by pressure from fluid in the lumen) might trigger a diameter increase in culture.

As a whole, our results back recent research suggesting that mechanical forces are key global coordinator of embryonic organ growth^35^ or growth inhibition^36^. Future investigations will be aimed at determining the signaling pathways^37,38^ responsible for sensing the mechanical forces and at determining their relation to biochemical actors involved in gut growth^39–41^. These investigations have important implications to understand organ growth in normal and pathological conditions (e.g. SBS, tumors), and also for the efficient design of organ regeneration methods.

## Materials and Methods

### Specimen preparation

Fertilized chicken eggs were purchased from EARL Morizeau (Chartres, France, breeder Hubbard, JA57 hen, I66 rooster, yielding type 657 chicks). The eggs were incubated at 37.5 °C in a humidified chamber for 8 to 10 days. The gastrointestinal tract was dissected out from the embryos from hindgut to proventrilicus.

### Culture with mechanical tension

The mesentery of the gut was carefully removed in PBS with tweezers because it prevented a clear assessment of morphological changes induced by culture; guts were laid flat by reducing the depth of PBS and photographed with a macroscope (Leica) in transmitted light. Small weights were obtained by cutting stainless steel pins (Euronexia, diameter 100–250 µm) to the required size and were weighed with a precision balance (Sartorius). These masses were attached to the distal most part of the hindgut; a full-length (4 cm) stainless steel pin was inserted in the stomach. The gut + pins were next transferred to a 15 mL plastic test tube filled with DMEM GlutaMAX^TM^-I (Thermoscientific, with 4.5 g/L D-glucose and sodium pyruvate) supplemented with 1% penicillin-streptomycin; the upper full-length pin rested on two wedges cut out at the tube opening; the lower pin (weight) pulled on the gut by gravity. Each gut was placed in an individual test tube; up to 8 guts were incubated simultaneously in a humidified incubator (Thermos) at 37.5 °C in a 5%CO_2_/95% air atmosphere. After 48 h, the guts were placed in individual Petri dishes in PBS at room-temperature, let to relax for 30 min and photographed. Guts were then further used to determine dry mass, histology or for dissociation assays.

### Determination of gut volume

Raw 2D images of the guts were processed to erase the hindgut, stomach and any residual tissue around the axially-symmetric midgut (segment comprised between caecal appendix tip to duodenum-stomach junction). We then thresholded these images and extracted the midgut contour (Matlab routine). We next applied a Voronoi algorithm^42^ to this contour to obtain the gut midline length and diameters along the midline. The volume is computed by integration of this data. We neglect the volume occupied by the lumen (<5% at E8 and E8+ 48 h culture, e.g. see Fig. 7a). We also resorted to a second, different procedure (Olaf plugin)^43^ which relies on manually fitting the gut contour with Bezier curves; the plugin automatically produces a 3D axisymmetric volume from the Bezier fit. We found that both methods yielded values of length, diameter and volume that were equal to within ~5%.

### Dry weight determination

We pulled heated Pasteur pipettes to obtain long (~10 cm), thin (~100 µm) glass fibers. The fibers were isolated from air currents using cardboard shields. We determined their sensitivity (°/N) in air by attaching 4 weights (nylon thread) of increasing, known mass, and by measuring the resulting angular deflection of the fiber with a camera (1600 × 1200 px Stingray FT-201, Allied Vision Technologies, equipped with a Computar macro lens x0.3-1). The deflection-force characteristic was linear (Fig. S7a); the slope is the fiber sensitivity. Water was removed from the guts by bathing them successively in 1:2, 2:1 and 1:0 absolute ethanol:water mixtures, for 30 minutes in each solution. This procedure warrants that all ethanol-miscible components like water, salts and any residual intraluminal fluid are washed away. The ethanol-soaked midgut (duodenum, jejunum and ileum) was then isolated with scissors and placed at the tip of the calibrated glass fiber. The final, stationary deflection angle after complete evaporation of the ethanol (Video S6) was measured and converted to mass using the previously determined fiber sensitivity. Dry weights could be measured with a precision of ±0.01 mg, which is an order of magnitude higher than what can be achieved with ordinary laboratory weighing scales. We found that the dry mass density of native E8 guts (*n* = 4, see Fig. S8a) is ${\rho }_{E8}=0.100\pm 0.015$ mg/mm^3^ (i.e. 10% dry mass, 90% water). This density was used to compute the mass of the guts before culture. The average density of unweighted guts after 2 day culture (*n* = 6) was ${\rho }_{ctl}=0.104\pm 0.016$ mg/mm^3^; the density of weighted guts (*n* = 10) had decreased on average by ~20% after 2 days in culture, ${\rho }_{tension}=0.081\pm 0.023$ mg/mm^3^. The dry mass density values we found are consistent with other measurements of dry mass of embryonic chick organ at similar stages^44^. Since we could not measure directly the dry mass before culture, we deduced it as ${\rho }_{E8}{V}_{i}$, where *V*_*i*_ is the measured volume of individual guts before culture. The dry mass change induced by culture (Fig. 5b) is $({m}_{f}-{\rho }_{E8}{V}_{i})/({\rho }_{E8}{V}_{i})$, where *m*_*f*_ is the measured dry mass of the gut after culture.

### Collagenase dissociation and cell-counting

The guts were enzymatically degraded for 10 min in 50 μL of a 2 mg/mL collagenase-dispase (Roche) solution in PBS at 37 °C. Each gut was then mechanically disrupted by up-and-down pipetting using a 100 µL Eppendorf tip until a homogeneous suspension was obtained. 50 μL of trypsin-EDTA (Thermofisher, 0.05%) was further added and the mixture was again pipetted to dissociate any residual cell aggregates. The suspension was stained with acridine orange/propidium iodide (AO/PI, MokaScience); this combination of fluorescent markers labels living cells in green and dead cells in red. The counting chamber volume is 0.5 μL. 600 nm (red) and 530 nm (green) fluorescence emission were recorded. Figure 5c shows a representative picture of cells in the counting chamber: the cells were uniformly dispersed, and had a sharply-peaked size distribution (SD = 0.3 μm). The average cell-size, percentage of living cells and cell count were automatically retrieved (Luna Cell Counter, Logos Biosystems) and averaged over 4 different images for each cell suspension. Following the same reasoning as for the determination of dry mass change, the cell number change induced by culture (Fig. 5e) is $({N}_{f}-{n}_{E8}{V}_{i})/({n}_{E8}{V}_{i})$, where *N*_*f*_ is the measured total cell number after culture, *n*_*E*8_ the measured average cell density of native E8 guts, and *V*_*i*_ the measured volume of the gut before culture. The average cell density of native E8 guts (*n* = 4) is ${{n}}_{{E}8}=4.15\,\pm \,{\mathrm{0.05.10}}^{5}$ cells/mm^3^; the average cell density of the guts after 2 day culture (*n* = 24) was higher by about ~50%, $6.60\,\pm \,{\mathrm{0.17.10}}^{5}$ cells/mm^3^. The cells in guts cultured without tension divide, leading to an increased total cell number, but this does not translate in a dry mass or net volume change, i.e. the unweighed guts do not grow. Consistent with these differences in cell density we also found that the average cell diameter was lower in cultured guts (6.3 ± 0.3 µm) than in native E8 guts (7.2 ± 0.5 µm) (Fig. 5d).

### Immunohistochemistry

Guts were fixed for 1 h in a 4% PFA in PBS solution, washed in PBS, then let overnight in 30% sucrose in water solutions, and embedded the next day in OCT compound (VWR) on dry ice. Thin (14 μm) slices were cut at −20 °C in a Leica cryotome and deposited on Thermofrost glass slides. After rehydration, the slides were blocked for 15 min in a 1% BSA and 0.1% triton in PBS solution, the slides were then incubated overnight in anti-α smooth muscle actin antibody (Abcam, ref5694, dilution 1:2000), anti βIII-tubulin antibody (Abcam, ref14545, dilution 1:1000) or anti-Histone H3 (phospho S10) (Abcam, ref14955, dilution 1:200) solution composed of 1% BSA in PBS; the following day, after washing, CY3- and A488-conjugated secondary antibodies (ThermoFisher, dilution 1:400 in PBS) were applied for 2 h. The slides were washed, sealed with a coverslip and immediately imaged with an epifluorescence or confocal microscope. Epithelial cells could be visualized in brightfield.

### Motility Analysis

The motility of the guts was assessed at select time points during culture by placing the culture tubes in a 37 °C water bath and by performing time-lapse imaging at 0.5 Hz frequency as described previously^23^. Motiligrams (=kymographs) were derived using the “Reslice” function of ImageJ. Constrictions in the guts that were tensed were shallower than those of the guts not subject to tension but their frequency did not differ (in the range 10–20 mHz for both unweighed and weighed guts, *n* = 3).

### Ethics Statement

The experiments were conducted under European law article 2016/63/UE. The approval of experimental protocols by an ethics committee is not required for research conducted on chicken at embryonic stages. All experiments were performed in accordance with the ethics guidelines of the INSERM and CNRS.

### Statistical analysis

Each group consisted of at least *n* = 6 samples, all samples are included in the presented data, no randomization or blinding was used, median, upper (75%) and lower (25%) interquartile range and means (empty square) are presented in Figs 5 and 6. All pairwise statistical analysis were performed using a two-tailed Mann-Whitney test. Differences were considered statistically meaningful at p < 0.05 (indicated by a star in Figs 5 and 6).

### Data and Code Availability

All data analyzed during this study are included in this published article (and its supplementary information files). The Matlab program developed for Voronoi analysis of the guts can be accessed within the context of a scientific collaboration.

## Electronic supplementary material

S6

S9

S10

Supplementary Information
