# Supplementary material for: Mechanical Tension Drives Elongational Growth of the Embryonic Gut
Source: Sci Rep. 2018 Apr 16;8:5995. doi: 10.1038/s41598-018-24368-1 (PMC5902462; doi:10.1038/s41598-018-24368-1)
Supplement: Supplementary file 4 — Supplementary Information [file 41598_2018_24368_MOESM4_ESM.pdf]

Supplementary Material to “Mechanical Tension Drives Elongational Growth of the Embryonic Gut“

Nicolas R. Chevalier, Tinke-Marie de Witte, Annemiek J.M. Cornelissen, Sylvie Dufour, Véronique Proux-Gillardeaux, Atef Asnacios

Explanatory Note S1:

The tensile force F applied by a mass m is $F = \frac{7}{8}mg$ where g is the standard gravity and the factor 7/8 accounts for buoyancy of the stainless steel mass in the liquid medium.

Figure S2:

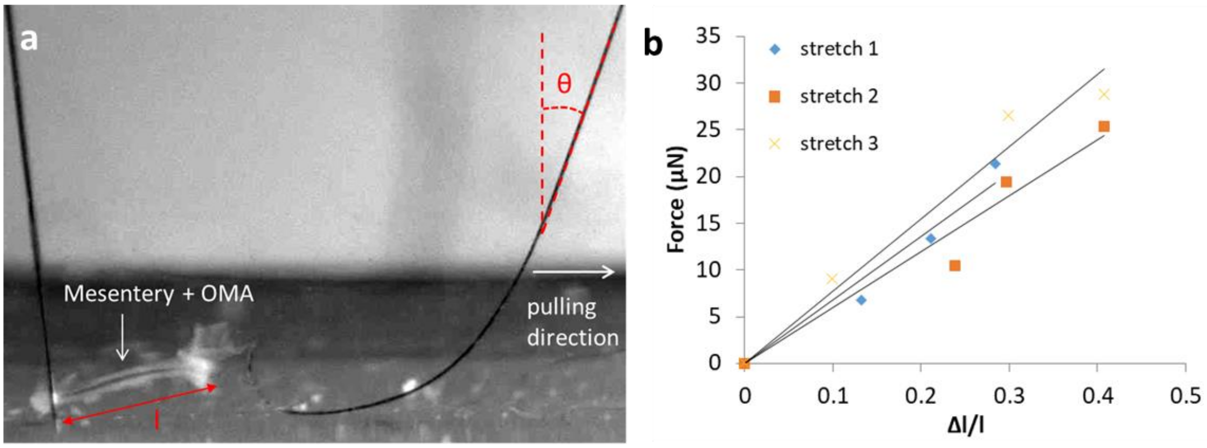

We isolated the mesentery + omphalomesenteric artery (OMA) located between the gut branches at stage E8 and performed stretch experiments in PBS at room temperature using a thin glass cantilever as depicted in Fig. S2a (the method is described in detail in [1]). The force is deduced from the deflection of the cantilever (sensitivity: 2.3 µN/°). In Fig.S2b, we show an example for three different stretches on the same sample of the force applied versus the elongation $\Delta l/l$ of the mesentery + OMA. The resistance to stretch is the slope of the lines in Fig.S2b. Each sample was stretched 3 times at a constant velocity (0.3 mm/sec), waiting 10 min between each stretch for the sample to relax back to its initial length. We found an average resistance of 60.2±10.6 µN (*n*=5 different samples, 15 stretches). We previously measured E_{tube}=1 kPa [2]; at E8, the gut diameter is $d_{tube}\sim$ 400 µm and the gut section $A_{tube} = \pi(d_{tube}/2)^2$. The resistance for the two gut segments is $2E_{tube}A_{tube} =$ 251µN. The fraction of the stress absorbed by the mesentery + OMA is therefore 60.2/251 = 23.9 %. We measured a physiological force on the gut loop of 9-17 µN, we therefore find that the force on the mesentery + OMA is 2.1-4 µN and the force on each gut branch is 3.4-6.6 µN.

Explanatory Note S3:

For our calculations, we consider the gut as a full cylinder. This assumption yields a negligible error on the computation of volume as the cross section of the lumen of native or cultured E8 guts was at most 5% of the total gut cross section (see for example sections in Fig.7c). In some rare cases we observed accumulation of fluid (DMEM) in the lumen in restricted segments of the midgut after culture which could lead to a local bulge – the bulge was erased when treating the image for Voronoi analysis and so are not taken into account in the volumes we report. The stress exerted by the mass m can be computed for a full cylinder of section S and elastic modulus E to be $\sigma = F/S$. The elastic stress at the level of the jejunum ($S \sim 0.1\ mm^2$) is 86 Pa for a 1 mg mass. Strain is the control parameter for most experiments studying the effect of tensile forces on cell cultures (using for example the FlexCell culture system, see [3,4]). Taking $E \sim 1\ kPa$ [2] an 86 Pa elastic stress yields an instantaneous elastic strain of 8.6%. The stress is slightly higher at the level of the ileum, which has a smaller section, and 2-3 times lower in the region of the caecal appendix, because the stress in this region is distributed over the section area of three different tubes (2 appendices and the ileum).

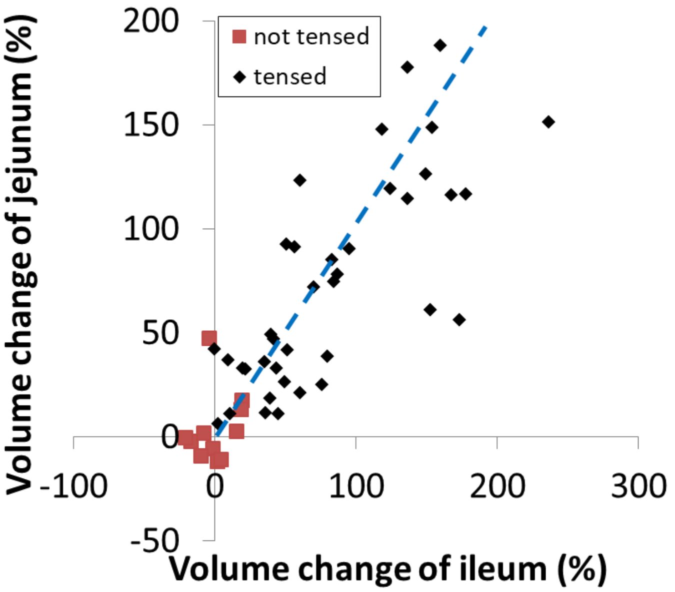

Figure S4: Volume change of the ileum versus volume change of the jejunum after 48 h culture, with (black diamonds) or without (red squares) tension; equal volume changes of these two segments is represented by a dashed blue line. The ileum and jejunum show similar volumetric growth.

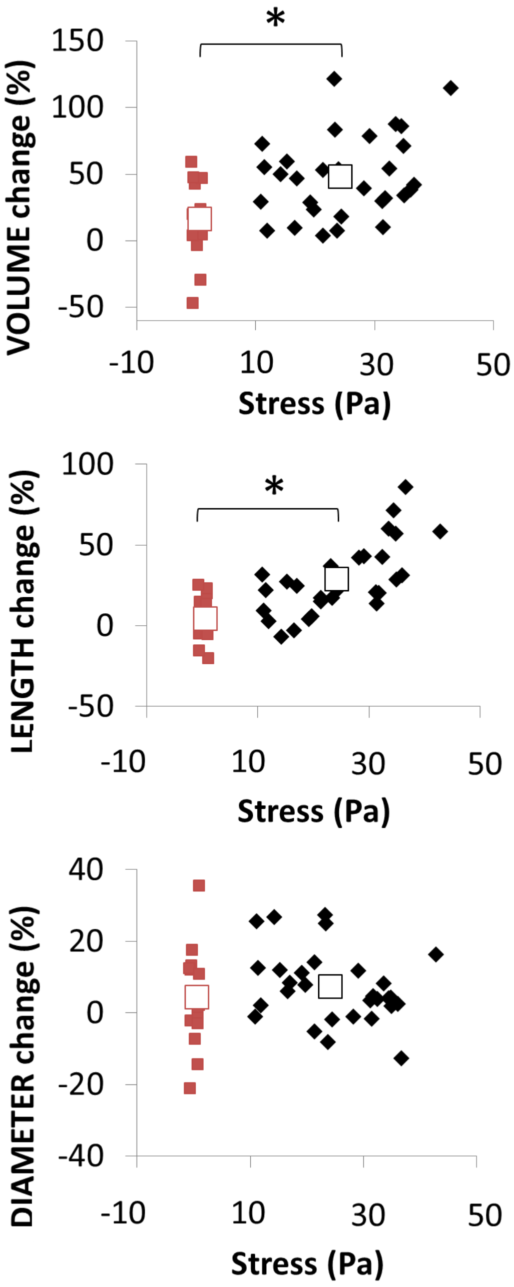

Figure S5: Volume, length and diameter change of caecal appendix before/after 48h culture without tension (red squares) or with tension (black diamonds). Average values are shown as empty squares. *: statistically significant difference, p<0.05, Mann Whitney two-tailed test. Each data point is a separate sample. The length l and average diameter d of a caecal appendix was measured using ImageJ line tool; the volume was deduced assuming a cylindrical geometry, $V = \pi l d^2/4$. The stress was computed assuming a local cross-section $3\pi d^2/4$, where d is the diameter of the caecal appendix, and the factor three takes into account the fact that the stress exerted by the hanging weight is distributed over the section area of three tubes (2 caecal appendices and the ileum). Unlike what was found for the midgut (Fig.4), the caecal appendices exhibited some basal level of volumetric growth (15±15%) even in the absence of tension. Growth of the caecal appendix of embryonic mouse guts cultured in the absence of continuous mechanical tension (catenary culture method) was also observed by Hearn et al.[2] Volumetric growth was however significantly higher with tension (47±14%).

Video S6: Example video of dry mass determination using a calibrated glass cantilever. The resting position of the cantilever is first determined, the ethanol soaked midgut is placed at the tip of the lever (the full cantilever length is about ~10 times the size of the segment seen on the movie). The ethanol eventually completely evaporates and the cantilever reaches a stationary, weighed position. The whole movie is then compressed to a single image using ImageJ > Z project > Max tool, an example is shown in Fig. S8b.

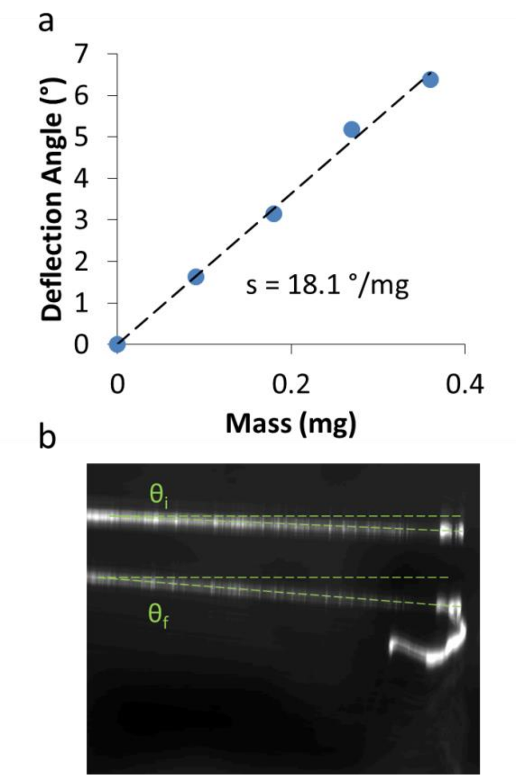

Figure S7: a) Calibration curve of the fiber cantilever using nylon wires of known mass. b) Image obtained from a weighing video (example: Video S7) using the "ImageJ > Z project > Max" tool from which the resting θ_i and weighed θ_f deflection angles of the cantilever are determined. The mass of the dry gut is $m_{dry} = (\theta_f - \theta_i)/s$. Uncertainty due to the determination of the deflection angle is ~0.2°, i.e., ~0.01 mg.

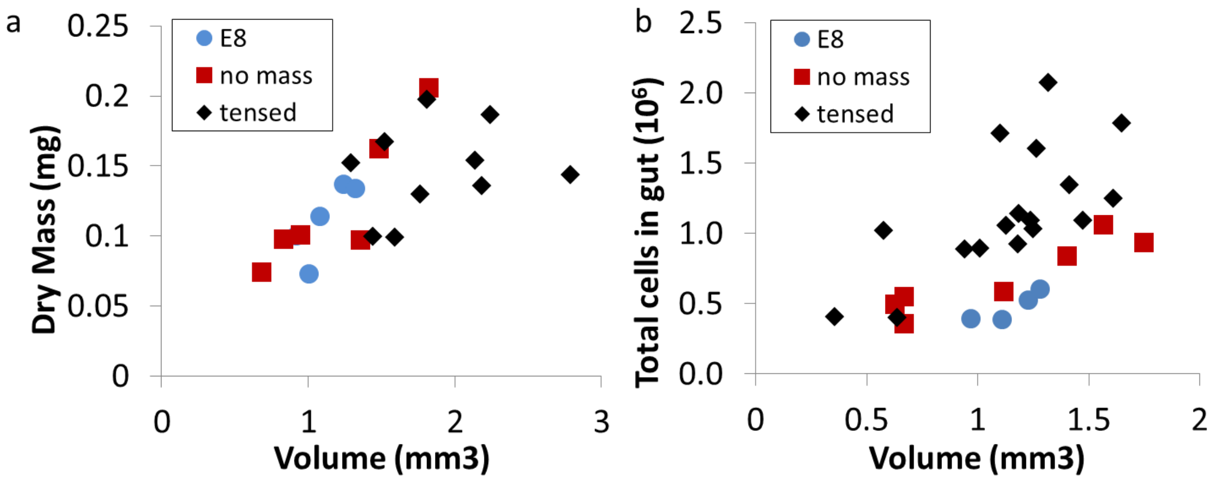

Figure S8: (a) Dry mass as a function of the volume of the guts after culture, without tension (red squares) or with tension (black diamonds). The measured dry mass and volumes of native E8 guts (blue dots) are also indicated. Error bar on mass determination of each sample is smaller than symbol size. (b) Cell number as a function of the volume of the guts after culture without tension (red squares) or with tension (black diamonds). The measured total cells and volumes of native E8 (blue dots) are also indicated. Error bar (SD) for each sample is smaller than symbol size.

Video S9: Time-lapse video of weighed (1 mg) gut from which the kymograph in Fig. 7c left was derived, after 1h culture. Propagating constrictions of the smooth muscle appear as dark, travelling bands.

Video S10: Time-lapse video of weighed (1 mg) gut from which the kymograph in Fig. 7c right was derived, after 48h culture.

References for Supplementary Material

[1] Chevalier, N. R.; Gazguez, E.; Dufour, S.; Fleury, V. Measuring the Micromechanical Properties of Embryonic Tissues. *Methods* **2016**, *94*, 120–128.

[2] N. R. Chevalier, E. Gazguez, L. Bidault, T. Guilbert, C. Vias, E. Vian, Y. Watanabe, L. Muller, S. Germain, N. Bondurand, S. Dufour, and V. Fleury, Sci. Rep. 6, 20927 (2016).

[3] B. Li, F. Li, K. M. Puskar, and J. H. Wang, 42, 1622 (2010).

[4] W. F. Liu, C. M. Nelson, J. L. Tan, and C. S. Chen, Circ. Res. 101, 44 (2007).
